# A combined method for DNA analysis and radiocarbon dating from a single sample

**DOI:** 10.1038/s41598-018-22472-w

**Published:** 2018-03-07

**Authors:** Petra Korlević, Sahra Talamo, Matthias Meyer

**Affiliations:** 1 Department of Evolutionary Genetics, Max Planck Institute for Evolutionary Anthropology, Leipzig, Germany; 2 Department of Human Evolution, Max Planck Institute for Evolutionary Anthropology, Leipzig, Germany

## Abstract

Current protocols for ancient DNA and radiocarbon analysis of ancient bones and teeth call for multiple destructive samplings of a given specimen, thereby increasing the extent of undesirable damage to precious archaeological material. Here we present a method that makes it possible to obtain both ancient DNA sequences and radiocarbon dates from the same sample material. This is achieved by releasing DNA from the bone matrix through incubation with either EDTA or phosphate buffer prior to complete demineralization and collagen extraction utilizing the acid-base-acid-gelatinization and ultrafiltration procedure established in most radiocarbon dating laboratories. Using a set of 12 bones of different ages and preservation conditions we demonstrate that on average 89% of the DNA can be released from sample powder with minimal, or 38% without any, detectable collagen loss. We also detect no skews in radiocarbon dates compared to untreated samples. Given the different material demands for radiocarbon dating (500 mg of bone/dentine) and DNA analysis (10–100 mg), combined DNA and collagen extraction not only streamlines the sampling process but also drastically increases the amount of DNA that can be recovered from limited sample material.

## Introduction

Over the past 70 years, radiocarbon dating has become an important tool for archaeology due to its precision in dating organic material up to approx. 50,000 years in age. The wide-spread use of radiocarbon dating has been facilitated by the use of accelerator mass spectrometry (AMS), which determines the ^14^C/^12^C ratio directly rather than measuring the release of beta particles from decaying ^14^C isotopes^1^. Carbon isotopes isolated from collagen are the primary source used in radiocarbon dating of bones and teeth, and current protocols require approximately 500 mg of bone or dentine with a minimum of 1% preserved collagen^2–4^.

More recently, advances in DNA sequencing technology have enabled the generation of genome-wide sequence data from hundreds of ancient remains, especially those of ancient humans^5–8^ and their extinct archaic relatives^9–11^, providing insights into the history of human groups, their dispersals and interactions. In contrast to AMS radiocarbon dating, genetic analysis of ancient bones and teeth is often feasible even from small amounts of sample material. This has been demonstrated, for example, in a series of genetic studies on fossil material from Denisova Cave, Russia. These included the recovery of high-quality genome sequences from a Neanderthal, as well as a Denisovan individual, a type of extinct hominin so far discovered only at this site, neither of which required more than 40 mg of bone material^9,10^. Useful genetic data was also retrieved from extremely small amounts of less well-preserved material^12–14^, most recently from as little as 10 mg of powder removed from a milk tooth discovered at the site, which was shown to belong to a Denisovan individual based on the analysis of 1 million base pairs of its nuclear genome^15^. Both destructive methods, DNA analysis and radiocarbon dating, are invaluable tools for reconstructing past events and their timing, such as the colonization of Europe by anatomically modern humans (AMH) and Neanderthal extinction^7,16–18^. However, the fossil record is often scarce and fragmentary, not only at Paleolithic sites, which limits the amount of material that can be sacrificed for molecular analyses. More importantly, every effort possible should be taken to keep destructive sampling to a minimum in order to preserve the world’s archaeological heritage for future generations.

Since carbonates in the mineral fraction of hard tissues are exchanged with those present in the environment^19^, it is necessary to completely remove the inorganic component of bone or dentine during collagen preparation for AMS radiocarbon dating. This is typically achieved by acid-base-acid (ABA) treatment^20^, in which a first treatment with hydrochloric acid solubilizes carbonates and hydroxyapatite, the main inorganic component of bones and teeth, a second treatment with sodium hydroxide removes other organic molecules such as humic acids, and a third treatment with hydrochloric acid removes atmospheric carbon dioxide absorbed during the base treatment. The resulting collagen is then incubated in acid at high temperature to produce soluble gelatine. Since carbon contamination may also arise from organic molecules that have entered the bone or tooth matrix through soil detritus, microbial invasion or post-excavation handling, ABA-gelatinization is often followed by ultrafiltration through membranes that separate high molecular weight collagen chains from shorter peptides, amino acids and other small molecules^3,21^.

DNA extraction, in contrast, is typically performed by lysis of the bone/tooth matrix using extraction buffers containing ethylenediaminetetraacetic acid (EDTA), a chelating agent that dissolves hydroxyapatite by means of sequestering calcium ions, and proteinase K, an enzyme that digests collagen and other proteins^22,23^. Even though the exact mechanism of DNA preservation in ancient bones and teeth is not fully understood^24^, the binding of negatively charged phosphate groups in the DNA backbone to positively charged calcium ions on the surface of hydroxyapatite crystals is thought to play a major role^25,26^. Therefore, the biomolecules required for radiocarbon dating and ancient DNA analysis are presumably located in different fractions of the bone matrix, suggesting that it might be feasible to retrieve both from a single sample by targeting the inorganic and organic components of the bone/tooth matrix separately. Such a combined method for DNA and collagen extraction would not only reduce the number of samplings and thereby the amount of material required to perform both techniques, but also substantially increase the amount of material available for genetic analyses.

Here we explored the feasibility of releasing DNA from ancient bones prior to collagen extraction using an ABA-gelatinization procedure followed by ultrafiltration. More specifically, we tested three reagents that might enable the recovery of DNA without degrading the organic component of the bone/tooth matrix. The first is EDTA, the reagent regularly used in ancient DNA extraction. EDTA is carbon-rich and synthesized from sources that contain only stable carbon isotopes (“old” carbon, ^12^C and ^13^C), and may skew dates to an older age if not properly removed^27,28^. The second reagent is a neutral (pH 7.0) sodium phosphate buffer. Phosphate buffers are commonly used in liquid chromatography^29^ and occasionally in ancient DNA research^30^ to release DNA from hydroxyapatite. Depending on their pH, they are composed of varying ratios of monosodium phosphate (NaH_2_PO_4_) and disodium phosphate (Na_2_HPO_4_), both of which are carbon-free. However, neutral phosphate buffers have been shown to preferentially release surface-bound microbial DNA rather than endogenous DNA from ancient bone^31^. We therefore tested as a third reagent the acidic monosodium buffer (subsequently referred to as ‘acidic phosphate’), which combines the release of DNA with mild demineralization of the bone matrix. For each of these reagents we evaluated the efficiency of DNA retrieval while monitoring possible losses of collagen and the accuracy of the resultant radiocarbon dates.

## Results

**Figure 1:**
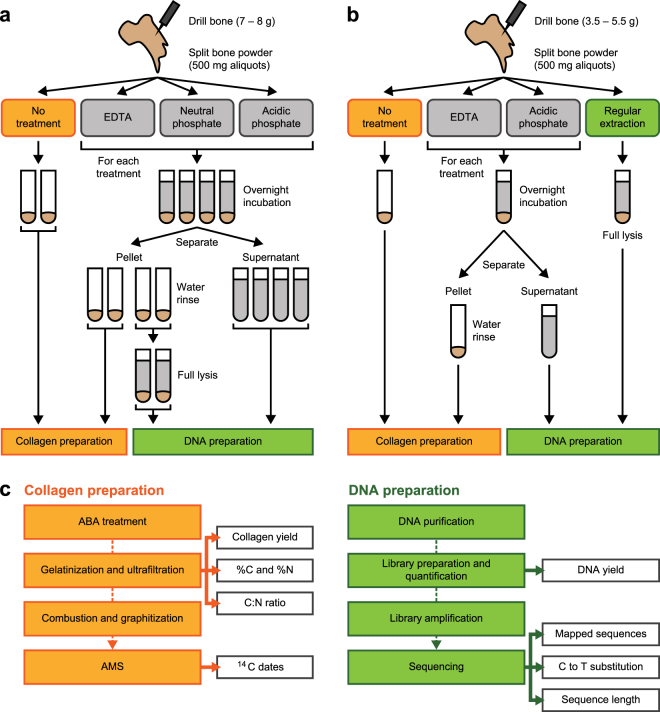
Schematic overview of the experiments performed in this study. (**a**) In an initial experiment, two bone samples were used to evaluate the suitability of EDTA as well as neutral and acidic phosphate buffers for releasing DNA prior to radiocarbon dating. (**b**) In a second experiment, 10 additional bones were used to determine the efficiency of DNA release with EDTA and acidic phosphate buffer, and the impact of these treatments on collagen preparation and radiocarbon dating. (**c**) Overview of the sample preparation workflows used for radiocarbon dating and genetic analysis.

To determine whether it is feasible in principle to extract DNA and collagen from the same sample material without affecting radiocarbon dates, we used a dentistry drill to remove 7 g of powder from a 300-year-old horse bone (sample A) close in age to the upper limit of radiocarbon dating, and 8 g of powder from a >50,000-year-old cave bear bone (“background bone”, sample B) containing no detectable endogenous ^14^C isotopes (see Table 1 and Supplementary Table S1 for details on the samples used in this study). The powder from each sample was split into 500 mg aliquots, which were then either subjected directly to collagen extraction and dating, or incubated with EDTA, neutral, or acidic phosphate buffers to release DNA (see Fig. 1a for a schematic overview of the experimental design). Following DNA release, half of the aliquots were used for collagen extraction and dating, and half were incubated with an EDTA/proteinase K buffer commonly used in ancient DNA extraction to achieve full lysis of the bone powder and release any residual DNA. DNA was isolated from the EDTA, phosphate and lysis buffers by silica-based purification and converted into DNA libraries. Yields of DNA library molecules were determined by digital PCR and the libraries characterized by high-throughput sequencing using Illumina’s MiSeq platform (Fig. 1c). Horse and cave bear DNA fragments (endogenous DNA) were identified by mapping sequences with a length of at least 35 base pairs (bp) to a closely related reference genome.

**Table 1:** Samples used in this study.

Sample	MPI DNA Code	MPI ^14^C Code	Species	Location	Preservation conditions	Previously estimated age
A	SP3885	R-EVA 616	Horse	Leipzig, Germany	Burial	1,644 AD (~300 BP) (context, ^14^C)^44^
B	SP3571	R-EVA 800	Cave bear	Schwabenreith Cave, Austria	Cave	>50 kBP (^14^C)*
C	SP1060	R-EVA 1656	Bottlenose dolphin	North Sea, Netherlands	Seabed	Early Holocene (context)
D	SP4052	R-EVA 123	Mammoth	Brown Bank – North Sea, UK	Seabed	~33 kBP (^14^C)^21^
E	SP4053	R-EVA 124	Woolly rhinoceros^†^	Brown Bank – North Sea, UK	Seabed	~43 kBP (^14^C)^21^
F	SP4054	R-EVA 572	Cave bear	Teixoneres Cave, Spain	Cave	Unit II 34–40 kBP (context)^45^
G	SP2689	R-EVA 1657	Cave bear	Vindija Cave, Croatia	Cave	~45 kBP (context)^46^
H	SP3391	R-EVA 1658	Yak	Denisova Cave, Russia	Cave	Layer 11.3 > 50 kBP (context)^12^
I	SP1421	R-EVA 1678	Mammoth	Bykovsky Peninsula – Siberia, Russia	Permafrost	~29 kBP (MKh-O468) (^14^C)^43^
J	SP3425	R-EVA 1679	Steppe bison	Yukon Territory, Canada	Permafrost	Unknown
K	SP4059	R-EVA 1680	Human	St. Lorenz by Schöningen, Germany	Burial	Late Middle Ages (context)
L	SP2513	R-EVA 1681	Dog	Netherlands	Burial	400 BC – 100 AD (context)

*Used as background bone (>50 kBP) in several radiocarbon dating laboratories.

^†^Previously assigned to bison, now re-assigned to woolly rhinoceros based on DNA analysis.

**Figure 2:**
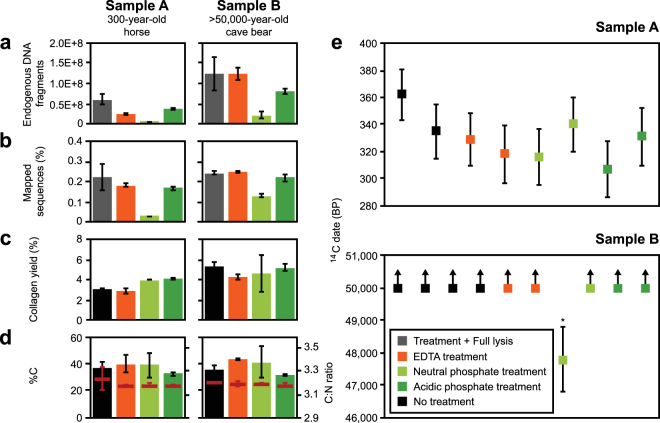
Comparing the suitability of EDTA, neutral and acidic phosphate treatments for DNA release prior to collagen extraction and radiocarbon dating. Plotted are (**a**) the number of endogenous DNA fragments recovered from the three reagents and full lysis of bone powder aliquots, (**b**) the percentage of DNA fragments that could be identified as endogenous by mapping to a reference genome, (**c**) the amount of collagen retrieved from bone powder expressed as the percentage of the starting mass, (**d**) the carbon content (%C) and C:N ratio (horizontal line) of the collagen preparation, and (**e**) uncalibrated AMS radiocarbon dates obtained from treated and untreated bone powder aliquots in years before present (BP). Error bars in panels a-d denote the standard deviation (±1σ) computed from technical replicates. Error bars in panel e indicate errors in AMS dating (±1σ). Outliers are marked with an asterisk (*).

By comparing the number of endogenous DNA fragments recovered during initial DNA release to those obtained from subsequent full lysis of the same bone powder aliquots, we estimate that EDTA released 42% and 99% of the endogenous DNA from samples A and B, respectively, while acidic phosphate released 53% and 50% (Fig. 2a and Supplementary Table S1). In contrast, no more than 20% of the endogenous DNA was released by incubation in the neutral phosphate buffer from either sample. DNA recovered from this buffer also showed a severe decrease in the relative abundance of endogenous vs. non-endogenous DNA fragments (Fig. 2b). While the size distributions of DNA fragments retrieved from EDTA and neutral phosphate were similar, acidic phosphate showed an enrichment for short DNA molecules (Supplementary Fig. S1). Prompted by these results we performed binding experiments of DNA to hydroxyapatite and bone powder, and found that when compared to long molecules, short molecules are both more efficiently released from hydroxyapatite by acidic phosphate and more efficiently retained from acidic buffers during subsequent silica-based DNA purification (Supplementary Figs S2 and S3).

Collagen yields from the powder aliquots used for DNA release are very similar to those that directly underwent collagen extraction (Fig. 2c). Likewise, the percentage of carbon and the carbon nitrogen ratios (C:N), which are routinely determined to assess the quality of collagen preparations^2,32^, were not substantially altered by DNA release (Fig. 2d). All collagen preparations from sample A produced consistent ^14^C dates, here defined as dates that fall into the combined 2σ interval with at least one of the untreated controls (95% confidence that the dates are identical within error)^33^, suggesting that no contamination with old carbon had occurred during DNA release (Fig. 2e). With the exception of one bone powder aliquot treated with neutral phosphate buffer, collagen extracted from sample B consistently yielded infinite dates. The single finite date (47,790 ± 1,000 BP) was obtained on the AMS directly after measuring a radiocarbon-rich sample, suggesting that a radiocarbon carryover might have brought the ^14^C concentration into detectable range (Supplementary Table S2). However, a phthalic acid (C_8_H_6_O_4_) blank that was measured on the same AMS magazine after a ^14^C-rich sample yielded an age of 53,000 BP, providing no evidence for cross-contamination. We also observed no detectable radiocarbon carryover in the phthalic acid and bone background blanks on the AMS magazine containing the radiocarbon-rich sample A. While it cannot be fully excluded that the observation in sample B was an isolated event of carbon carryover on the AMS, it is also possible that contamination with small amounts of modern carbon occurred during DNA release or collagen preparation.

As the results of the first experiment were in principle encouraging, we applied the two most effective strategies for DNA release, pretreatment of bone powder with EDTA and acidic phosphate buffer, to a set of 10 bones (samples C-L) in order to determine if these methods produce consistent results when applied to materials of various ages and preservation conditions (caves, burials, seabed and permafrost) (Table 1 and Supplementary Table S1). The experimental design was similar to the previous one, except that the DNA release was determined by comparing the number of DNA fragments released in the treatments to those released by full lysis of a separate powder aliquot (Fig. 1b).

**Figure 3:**
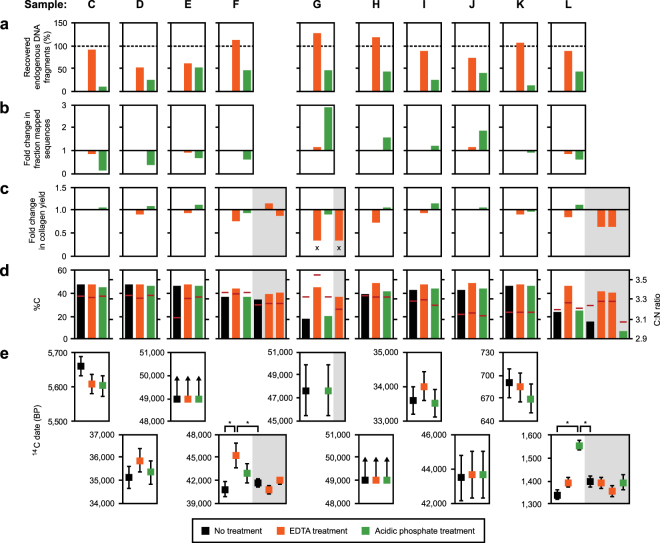
Combining DNA and collagen extraction on 10 bones of various ages. Plotted are (**a**) the number of endogenous DNA fragments released with EDTA and acidic phosphate treatment relative to the amount retrieved from a full lysis of untreated powder, (**b**) the change in the fraction of mapped sequences relative to untreated powder, (**c**) the amount of collagen recovered after treatment relative to untreated powder, (**d**) the carbon content (%C) and C:N ratio (horizontal line) of the collagen preparation, and (**e**) the uncalibrated AMS radiocarbon dates obtained from treated and untreated bone powder in years before present (BP). Error bars in panel e indicate errors in AMS dating (±1σ). Outliers are marked with an asterisk (*). Samples with insufficient collagen yields for dating are marked with an ‘x’. Technical replicates are shaded in grey.

Averaged across the 10 bones, the release of endogenous DNA was estimated to be 93% for EDTA (with a minimum of 53%) and 36% for acidic phosphate (ranging from 11% to 52%), showing that an incubation with EDTA enables retrieval of nearly all of the endogenous DNA present in these specimens (Fig. 3a and Supplementary Table S1). Averaged across all samples, the ratio of endogenous to non-endogenous DNA obtained after each of the two treatments was similar to that obtained by full lysis of the untreated control sample powder. However, if samples are considered individually, the percentage of endogenous DNA obtained by acidic phosphate treatment varies substantially when compared to the untreated control, ranging from an 8.3-fold decrease in sample C to a 2.9-fold increase in sample G (Fig. 3b). This may be partially driven by differences in the size of DNA fragments recovered with acidic phosphate, which are shorter in most samples (Supplementary Fig. S4), consistent with the results of the previous experiment. For bone powder aliquots treated with EDTA, we observed a 17% reduction in collagen yield on average (ranging from a loss of 67% to a gain of 2%) compared to untreated bone powder (Fig. 3c). Loss of collagen is mostly driven by a single sample (sample G), where insufficient yield after EDTA treatment compromised our ability to date the material. This result was reproduced when repeating EDTA treatment and collagen extraction for this sample. Acidic phosphate, on the other hand, did not reduce collagen yields. Interestingly, the carbon content was generally higher in the EDTA-treated powder aliquots (Fig. 3d), suggesting that EDTA treatment may have improved the quality of the collagen preparation, especially in samples that showed the strongest reduction in collagen yields, although one of the EDTA-treated powder aliquots from sample G produced a C:N ratio of 3.5, close to the upper end of the acceptable range. We also compared the amount of collagen and the number of endogenous DNA fragments recovered from all 12 samples analysed in this study but found no significant correlation (Pearson’s Correlation Coefficient: R = 0.4076, p = 0.1884; Supplementary Fig. S5).

AMS radiocarbon dates of most treated aliquots were consistent with those obtained from the untreated controls (Fig. 3e). We did, however, observe two outliers, both of which were shifted towards slightly older dates: the first was produced by the EDTA-treated powder of sample F, one of the oldest bones in the set, and the second belonged to the phosphate-treated powder of sample L, the second youngest bone in the set. These results indicate that no modern ^14^C isotopes were introduced during DNA release, instead they point to a possible contamination with “old” ^14^C-free carbon. Interestingly, the highest outlier was produced by bone powder treated with phosphate buffer, a reagent that may conceivably be contaminated by modern carbon from atmospheric carbon dioxide, but should be free of old carbon. Furthermore, even if old carbon from an unknown source had been introduced during DNA release, dates from young samples would be expected to be particularly strongly affected by such contamination, a pattern not seen in our data.

To evaluate whether outliers appeared stochastically in our data set, we repeated the DNA release and dating for samples F and L (Fig. 3e). This time, all dates were consistent with the untreated controls. Given that a total of 54 dates were generated in the course of our experiments, the occurrence of three outliers (in samples B, F, and L) is not unexpected, and indicates that DNA release, collagen preparation and graphitization together contributed only small and unsystematic error to the dating process. We thus conclude that DNA release using EDTA or acidic phosphate buffer has no detectable effect on the accuracy of radiocarbon dating.

## Discussion

By simultaneously recovering DNA sequences and radiocarbon dates from 12 ancient bones, we have successfully demonstrated that substantial amounts of DNA can be released from sample powder prior to radiocarbon dating without reducing the accuracy of dating. We identified two methods that are suitable for this purpose. The first, incubation of bone powder with EDTA, releases nearly as much DNA from the sample as can be obtained by full lysis of the bone matrix during regular DNA extraction. However, decalcification and DNA release with EDTA, when coupled with ABA-gelatinization treatment, comes at the expense of collagen yields. These losses in collagen are small in most cases, but can occasionally reduce collagen yields below the minimum amount required for dating. The second method, which relies on pretreatment of bone powder with an acidic phosphate buffer, has no detrimental effect on collagen preparation with the ABA-gelatinization procedure, but releases only between 10 and 50% of the DNA from the sample powder. However, if 500 mg of material is used for dating, this corresponds to an amount of DNA that would otherwise require removing an additional 50–250 mg of material exclusively for genetic analysis, which is substantially more than what is typically used in many studies involving precious specimens. Releasing DNA with acidic phosphate prior to collagen preparation thus not only avoids the need for additional destructive samplings of the bone/tooth material, but also drastically increases the amount of genetic information that can be retrieved from precious fossil material. This approach may indeed be the only way to retrieve both radiocarbon dates and genetic information from extremely small specimens, for example skeletal remains of small vertebrates or individual hominin teeth, where modified ABA-gelatinization and AMS measurement strategies have to be applied to attempt dating from very small (<100 mg) amounts of bone^34,35^.

While the reduced need for destructive sampling is the most obvious benefit of a combined collagen and DNA extraction workflow, it may also help to obtain samples of higher quality for DNA analysis. The reconstruction of whole genome sequences from ancient bones and teeth is often hampered by contamination with microbial, human and other environmental DNA. This problem can in some instances be alleviated by enriching for parts of the genome by hybridization capture^36^ or restricting analyses to DNA fragments that carry ancient DNA specific base damage^37,38^. These enrichment strategies can also be applied to DNA that was released from bone or dentine powder prior to collagen extraction. However, it has also been shown that DNA preservation and contamination with exogenous DNA can vary greatly within one specimen, even in locations that are in close proximity. Extracting DNA from several sampling spots can thus help in obtaining DNA extracts that are richer in endogenous DNA and less contaminated, improving the scope of genetic analysis that can be performed on a given sample^39^. As material demands for radiocarbon dating are large, powder can be collected in multiple small sub-samples (e.g. 10 samples of 50 mg each) instead of removing a single large sample. DNA can then be released separately from these sub-samples before combining them prior to the ABA-gelatinization procedure for collagen extraction. We currently recommend that the DNA release step is performed in the radiocarbon dating laboratory not more than a week before entering the ABA procedure as further work is needed to determine the long-term stability of samples after DNA release.

It is important to note that our work focused exclusively on methods that are compatible with collagen extraction using the well-established ABA-gelatinization procedure. A less commonly used method for collagen preparation relies on decalcification of the bone matrix using EDTA instead of strong acids^28,40^. While it has been suggested that omitting the acid and base treatments prior to gelatinization in collagen preparation increases the yield of collagen^41,42^, disadvantages of this approach are the longer times required for complete decalcification and the possibility of skewing radiocarbon dates towards older ages^27^. However, it has been shown here and previously^28^ that accurate dates can be obtained if EDTA is properly removed after decalcification. The loss of collagen we observed with EDTA treatment is likely the result of repeated decalcification during DNA release and subsequent ABA-gelatinization. In fact, our data suggests that the quality of isolated collagen may be higher with EDTA decalcification. Considering the substantially higher DNA yields obtained by EDTA treatment of bone powder compared to acidic phosphate, it seems that an EDTA-only collagen preparation might offer a more straight-forward and efficient approach for combining DNA analysis and radiocarbon dating. For the few radiocarbon dating laboratories that are already relying on EDTA decalcification for collagen preparation, this requires nothing else than storing the EDTA fraction for future DNA analysis. For laboratories using the ABA-gelatinization procedure, pretreatment of bone powder with acidic phosphate provides a less efficient but safer method for releasing DNA from precious sample material prior to collagen preparation and radiocarbon dating.

In summary, we have shown that two important ancient biomolecules, DNA and collagen, can be recovered from the same sample material. We hope that the work presented here will stimulate further research towards a deeper integration of the sample preparation workflows used for molecular analysis of ancient skeletal remains, leading to minimal destructive sampling of precious archaeological material.

## Methods

### Samples

A total of 12 bone samples from various species (bison, cave bear, dog, dolphin, horse, human, mammoth, woolly rhinoceros, yak), preservation conditions (cave, burial, permafrost, seabed) and ages (from 300 to > 50,000 ^14^C BP) were used in this research (Table 1 and Supplementary Table S1). Five samples were previously directly radiocarbon dated^21,43,44^, while the age of the other seven specimens was unknown or inferred from their chronological context^12,45,46^. Sampling was performed in a designated ancient DNA cleanroom. From each sample a large amount of bone powder (3.5–8 g) was drilled using a sterile dentistry drill at lowest speed to avoid heating, and the powder was split into approximately 500 mg aliquots for each treatment. Leftover powder was stored at room temperature for further use if needed.

### Pretreatment, DNA extraction, library preparation and sequencing

Following the experimental design summarized in Fig. 1, 500 mg bone powder aliquots of samples A and B (first experiment) and samples C-L (second experiment) were transferred into either bleach and UV decontaminated 12 mL screw cap borosilicate glass tubes (Kimble Chase Life Science) (for untreated controls and aliquots for acidic phosphate treatment) or Falcon 15 mL conical centrifuge tubes (Thermo Fisher Scientific) (for EDTA and neutral phosphate treatment). To these aliquots, 10 mL of either 0.5 M EDTA pH 8.0 (AppliChem), 0.5 M sodium phosphate pH 7.0 (Alfa Aesar) or 0.5 M monosodium phosphate pH 3.0–4.5 (5 M stock solution supplied by Sigma-Aldrich) were added, and the bone powder suspension was rotated at room temperature overnight (approx. 22 h). Bone powder was pelleted by centrifugation and the supernatants pipetted into new 15 mL Falcon tubes. The EDTA-treated bone pellets were then washed 5 times, and the phosphate-treated pellets 3 times, by adding 10 mL water, resuspending the powder by vortexing, pelleting the powder in a centrifuge and removing the supernatant. After the final resuspension in water, bone powder aliquots devoted to radiocarbon dating were transferred from the ancient DNA cleanroom to the radiocarbon preparation laboratory at the MPI-EVA and stored at 4 °C for up to a week prior to removing the supernatant and continuing with ABA treatment. DNA was extracted from treated (first experiment) and untreated (second experiment) bone powder aliquots by adding 10 mL of lysis buffer (0.45 M EDTA, 0.25 mg/ml proteinase K, 0.05% Tween-20), vortexing, and incubating with rotation at 37 °C overnight. After pelleting the remaining bone powder by centrifugation, the supernatant was pipetted into a fresh tube and 1 mL of lysis buffer was used for DNA purification using a protocol optimized for silica columns^47^ with modifications^31^. The remaining 9 mL lysis buffer was stored at -20 °C for future usage. DNA was purified from the EDTA, neutral and acidic phosphate buffers using 1 mL of each reagent and following the same protocol.

DNA libraries from the experiment which included samples A and B were prepared from 10 µL aliquots of DNA extract (total extract volume of 50 µL) using CircLigase-based, single-stranded library preparation^48^ with modifications^31^. Extracts from the remaining samples were converted into DNA libraries using a more recent implementation of single-stranded library preparation^49^ automated on a Bravo NGS Workstation (Agilent Technologies)^50^. Negative controls (buffers containing no sample powder or DNA extract) were included during the initial bone powder treatment, DNA extraction and library preparation, and carried alongside the samples throughout all experiments. The total number of molecules in each library was measured by digital PCR as described elsewhere^31^. Libraries were then amplified to PCR plateau and tagged with two sample-specific indexes^51^ using AccuPrime *Pfx* DNA polymerase^52^ and 1 µM of each indexing primer. Libraries were pooled and heteroduplexes removed by a single PCR cycle using Herculase II Fusion DNA Polymerase^52^ and IS5/IS6 primer pairs^53^. DNA concentrations in the library pools were measured on an Agilent Technologies Bioanalyzer 2100 using DNA-1000 chips.

Library pools were diluted and sequenced on Illumina’s MiSeq or HiSeq 2500 platform using a 76 + 7 + 76 + 7 cycle recipe^51^. Base calling was performed using Illumina’s Bustard software. Forward and reverse reads were overlap-merged to reconstruct full-length sequences^54^ and assigned to the parent library based on perfect matches to expected index combinations. Where necessary, species identity of samples was assessed by analysing sequences mapping to mammalian mitochondrial genomes^55^. Libraries were then aligned to appropriate reference genomes (cow, dog, dolphin, elephant, horse, human (hg19), polar bear and rhinoceros) using BWA^56^ with ancient DNA parameters^9^. Sequences shorter than 35 bp were discarded and PCR duplicates were removed by calling a consensus from sequences with the same alignment start and end coordinates (bam-rmdup; https://bitbucket.org/ustenzel/biohazard-tools). Summary statistics were computed using custom Perl scripts^49^.

In order to evaluate the suitability of our silica-based DNA purification protocol^47^ for DNA retrieval from all buffers used in this study, a mixture containing 1 μg (2 μL) of Thermo Scientific GeneRuler Ultra Low Range DNA Ladder, 500 µL of guanidine hydrochloride binding buffer and 20 µL of 3 M sodium acetate was added to 100 µL of either water, TET buffer (10 mM Tris-HCl, 1 mM EDTA, 0.05% Tween-20), 0.5 M EDTA pH 8.0, 0.5 M sodium phosphate pH 7.0, 0.5 M monosodium phosphate pH 3.0–4.5, lysis buffer, or 0.5 M sodium acetate pH 5.2, and purified using MinElute silica columns. Following separation of DNA extracts on a 4% agarose gel, we confirmed that DNA fragments ≥ 35 bp were efficiently retrieved from all buffers, while DNA fragments between 20–25 bp were also retrieved from water, TET, monosodium phosphate and sodium acetate, pointing to a better recovery of short fragments from low-salt or acidic solutions (Supplementary Fig. S3).

To assess the efficiency of all pretreatment buffers for releasing DNA bound to bone matrix, 40 µL (20 µg) of the same DNA ladder was bound to 160 mg of either hydroxyapatite or bone powder, washed with water and split into aliquots following a previously described procedure^31^. Each aliquot was then incubated for three hours with rotation at room temperature in 1 mL of either EDTA, neutral phosphate or acidic phosphate. After centrifugation, the supernatants were transferred to new tubes, the pellets were washed with water and incubated overnight with rotation at room temperature in 1 mL of lysis buffer to release any remaining ladder DNA (lysis buffer incubation after EDTA pretreatment was omitted for hydroxyapatite as it dissolved during the three hour incubation). Buffers were desalted twice with TE buffer (10 mM Tris-HCl, 1 mM EDTA) using Amicon Ultra-4 3 K Centrifugal Filter Units (Millipore) following the manufacturer’s protocol, and DNA was separated and visualized on a 4% agarose gel. All three pretreatment buffers were able to release the full range of DNA fragments from bone powder with high efficiency, while acidic phosphate preferentially released shorter (≤75 bp) fragments from hydroxyapatite (Supplementary Fig. S2).

### Collagen preparation and AMS measurement

Collagen was extracted for both radiocarbon and isotopic analyses following a previously established pretreatment protocol^21^. EDTA and neutral phosphate treated bone powder aliquots were transferred from Falcon tubes into 12 mL screw cap borosilicate glass tubes for subsequent ABA treatment. All samples were decalcified in 0.5 M HCl at room temperature until no CO_2_ effervescence was observed (from 1 day up to 1 week, with an HCl refresh for longer incubations). The acid treated portion was then rinsed with Milli-Q water and immersed in 0.1 M NaOH for 30 min to remove humics. The NaOH step was followed by a final 0.5 M HCl incubation for 15 min. The resulting solid was gelatinized at pH 3 in a heater block set to 75 °C for 20 h^20^. To remove contaminants, the gelatine was first filtered in an Eeze-Filter™ (Elkay Laboratory Products (UK) Ltd.) to remove small (>80 μm) particles, and then ultrafiltered^57^ using Sartorius”VivaspinTurbo” 30 KDa ultrafilters. Prior to use, the filters were cleaned to remove carbon-containing humectants^58,59^. The samples were lyophilized for 48 h.

To assess the preservation and amount of obtained collagen, C:N ratios and isotopic values were evaluated. Based on present-day samples, the C:N ratio should be between 2.9 and 3.6, while the collagen yield should not be less than 1% of the starting weight^2,32^. Stable isotopic analysis were evaluated at MPI-EVA, Leipzig (lab code: S-EVA), using a ThermoFinnigan Flash EA coupled to a Delta V isotope ratio mass spectrometer.

For samples that passed the collagen evaluation criteria, between 3 and 5 mg of collagen was weighed into pre-cleaned tin cups at the MPI-EVA and sent to the Klaus-Tschira-AMS facility (lab code: MAMS). The sample was combusted in an EA, and CO_2_ was converted catalytically to graphite, which was dated using a MICADAS-AMS^60^. All dates were corrected for a residual preparation background estimated from ^14^C-free bone samples provided by the Mannheim laboratory and pretreated in the same way as the samples studied here. Radiocarbon dates from treated and untreated powder aliquots for each sample, excluding outliers, were averaged and calibrated in OxCal v4.2^61^ using calibration curves Marine13 for sample C and IntCal13 for all other samples^62^ (Supplementary Table S1).

### Data Availability

All data generated or analysed for this study are included in the submitted manuscript (and its Supplementary Information files). Additional information, such as full DNA shotgun sequencing data, is available from the corresponding author on request.

## Electronic supplementary material

Supplementary Information

Supplementary Dataset
