# Supplementary material for: A combined method for DNA analysis and radiocarbon dating from a single sample
Source: Sci Rep. 2018 Mar 7;8:4127. doi: 10.1038/s41598-018-22472-w (PMC5841407; doi:10.1038/s41598-018-22472-w)
Supplement: Supplementary file 1 — Supplementary Information [file 41598_2018_22472_MOESM1_ESM.pdf]

# Supplementary Information

## A combined method for DNA analysis and radiocarbon dating from a single sample

Petra Korlević[1], Sahra Talamo[2]§, and Matthias Meyer[1]§

*[1]Department of Evolutionary Genetics, Max Planck Institute for Evolutionary Anthropology, Leipzig, Germany*

*[2]Department of Human Evolution, Max Planck Institute for Evolutionary Anthropology, Leipzig, Germany*

*§ These authors contributed equally to this work*

**This file includes:**

Supplementary Figures S1 – S5

Supplementary Table S2

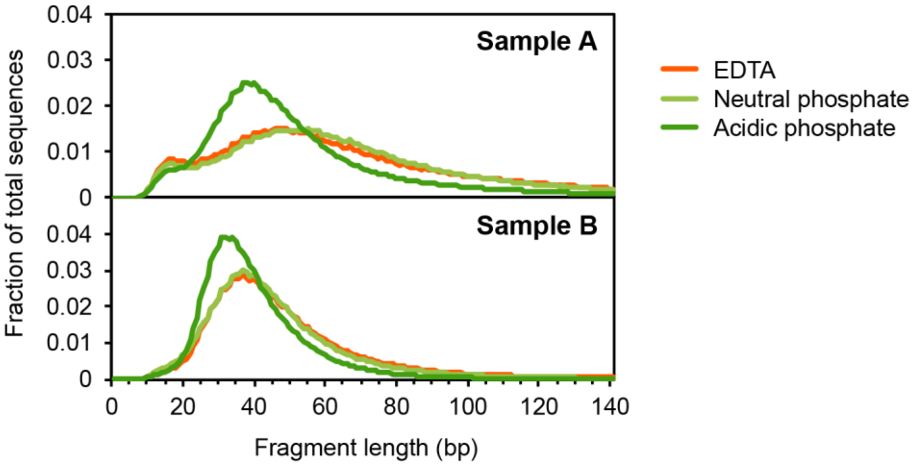


**Supplementary Figure S1.** Effect of different extraction methods on the size distribution of DNA fragments for sample A (300-year-old horse bone) and sample B (>50,000-year-old cave bear bone) using the length of all overlap-merged sequences. Data from technical replicates were combined.

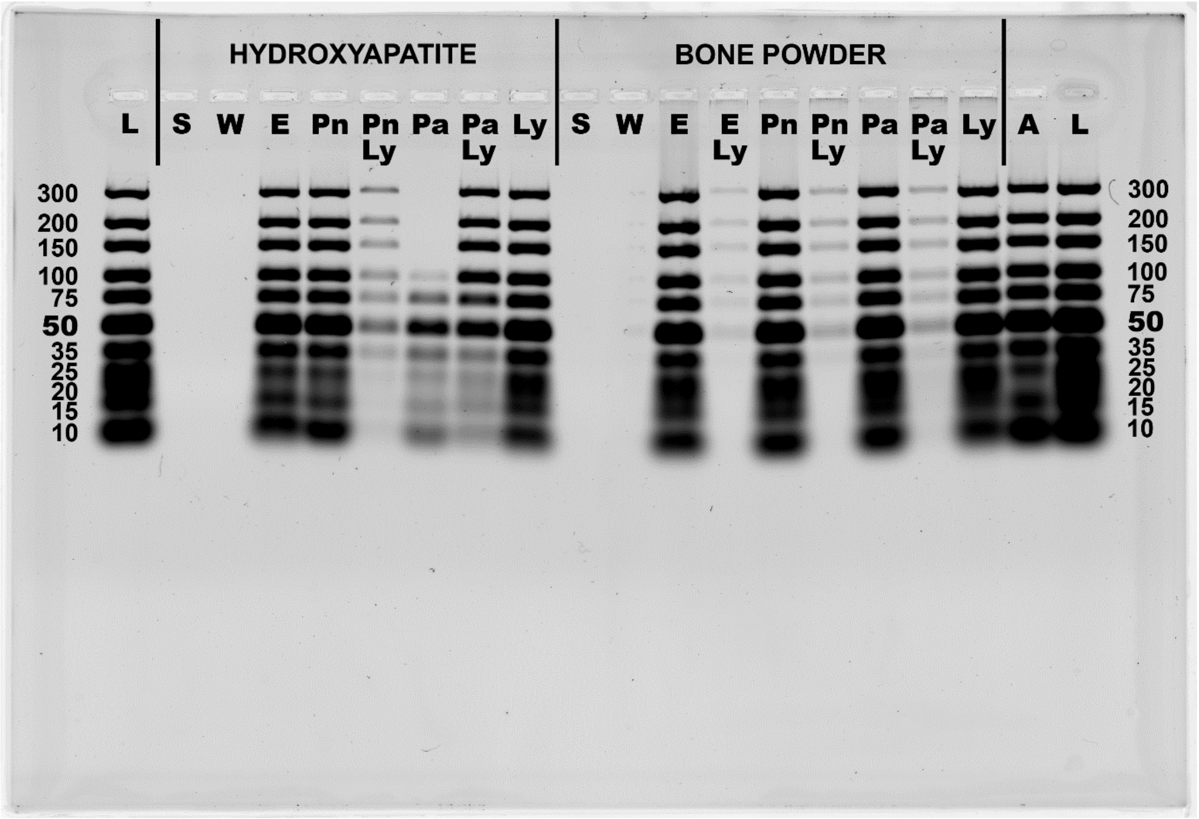


**Supplementary Figure S2.** Efficiency of DNA release from hydroxyapatite and ancient bone powder. **(L)** Thermo Scientific GeneRuler Ultra Low Range DNA Ladder, **(S)** supernatant after DNA binding, **(W)** first water wash after DNA binding, **(E)** EDTA incubation, **(E-Ly)** lysis buffer incubation after E, **(Pn)** neutral phosphate incubation, **(Pn-Ly)** lysis buffer incubation after Pn, **(Pa)** acidic phosphate incubation, **(Pa-Ly)** lysis buffer incubation after Pa, **(Ly)** regular lysis buffer incubation without prior treatment, **(A)** Amicon purification control (10 µL DNA ladder in water). Single agarose gel photographed using a Typhoon 9410 Variable Mode Imager and labelled using Adobe Photoshop with minimal processing.

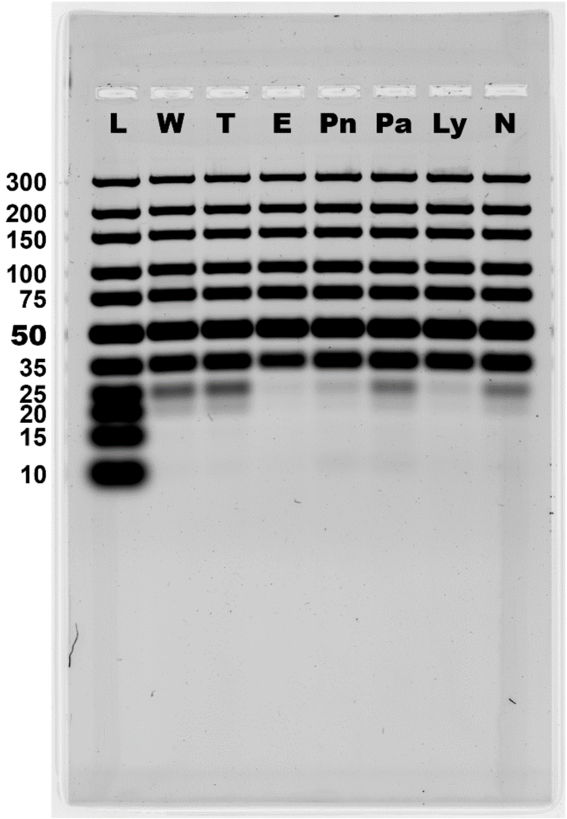


**Supplementary Figure S3.** Efficiency of DNA retrieval from various buffers using silica-based DNA extraction. **(L)** Thermo Scientific GeneRuler Ultra Low Range DNA Ladder, **(W)** water, **(T)** TET buffer, **(E)** 0.5 M EDTA, **(Pn)** 0.5 M sodium phosphate (‘neutral’), **(Pa)** 0.5 M monosodium phosphate (‘acidic’), **(Ly)** lysis buffer, **(N)** 0.5 M sodium acetate. Single agarose gel photographed using a Typhoon 9410 Variable Mode Imager and labelled using Adobe Photoshop with minimal processing.

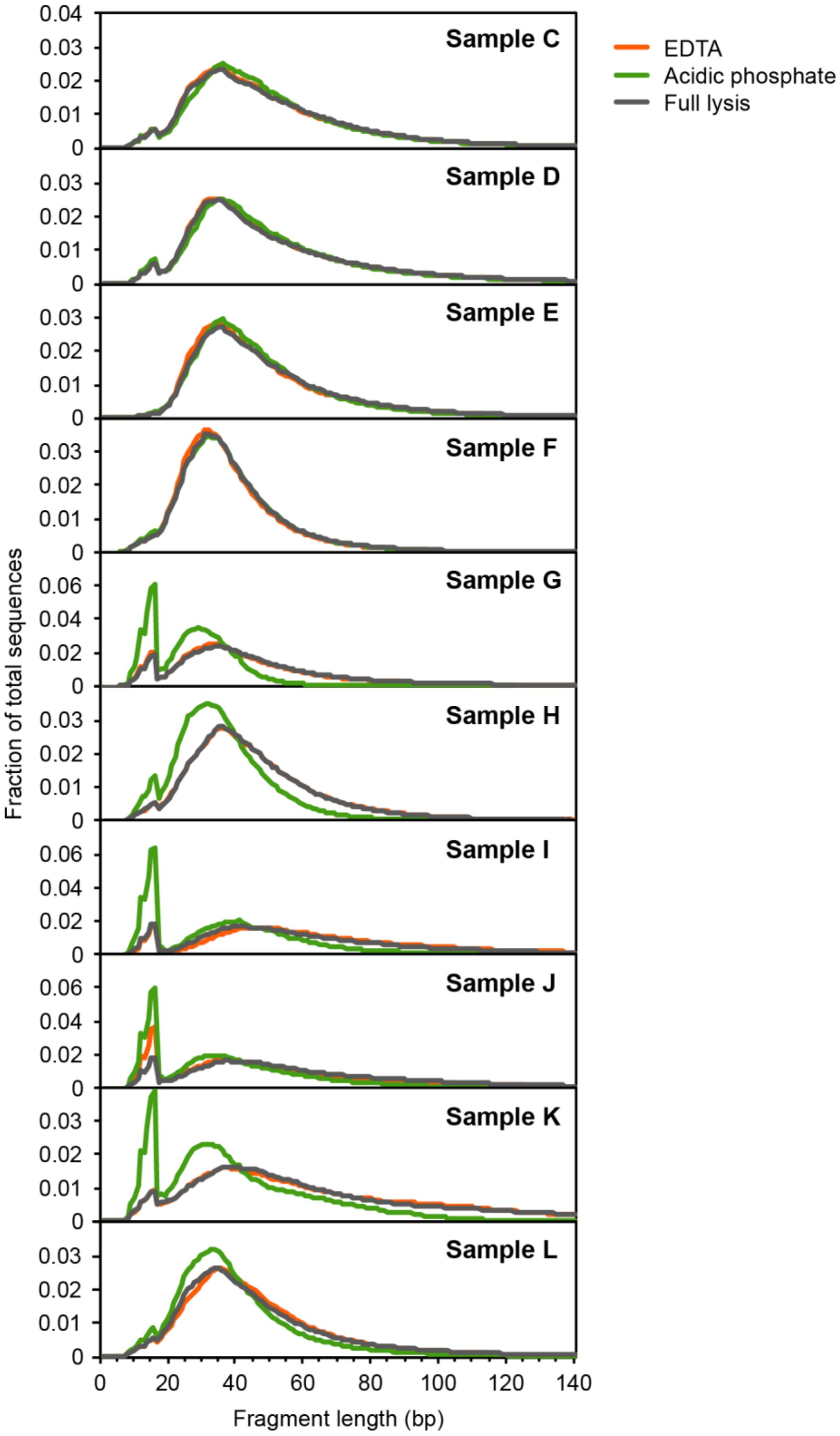


**Supplementary Figure S4.** Effects of DNA release with EDTA and acidic phosphate on the size distribution of DNA fragments recovered from samples C-L using the length of all overlap-merged sequences. Peaks around 16-18 bp in some libraries represent artefacts from library preparation.

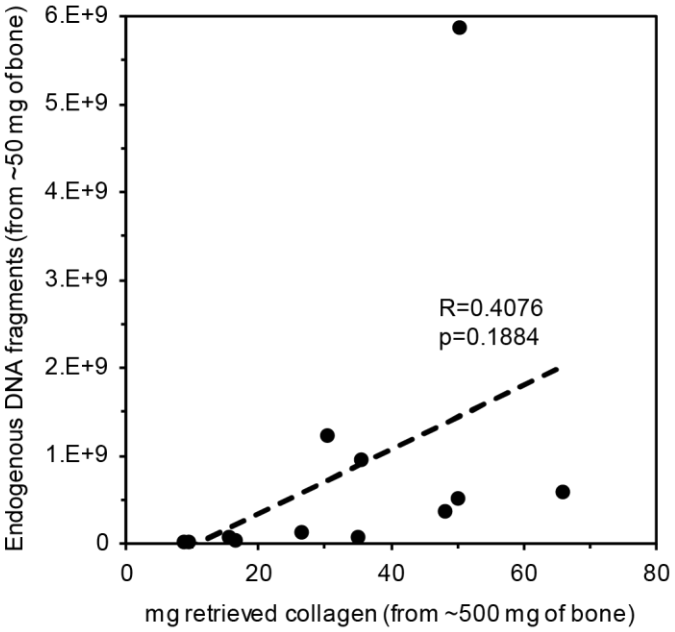


**Supplementary Figure S5.** Correlation between the amount of endogenous DNA retrieved by full lysis of bone powder and the amount of collagen retrieved from untreated bone powder. Each dot represents one of the 12 bone samples used in this project, with a dashed line representing the linear fit. Statistical analysis was performed using the web-interface Pearson's Correlation Coefficient calculator at Social Science Statistics (http://www.socscistatistics.com).

**Supplementary Table S2.** Raw $F^{14}C$ measurements (fraction of $^{14}C$ isotopes in a sample relative to 95% of the $^{14}C$ activity of Oxalic Acid I measured in 1950) from AMS magazines MA150713, which contained one part of the measurements for sample B (>50,000-year-old cave bear), and MA160114, which contained all measurements for sample A (300-year-old horse). Background measurements from phthalic acid and bone blanks for both magazines are also shown. An $F^{14}C$ value close to 1 denotes an age of 0 BP, while $F^{14}C$~0 denotes an age older than 50,000 BP.

| Magazine № | MAMS № | Sample | $^{14}C$ age ±1σ (BP) | $F^{14}C$ ±1σ |
|---|---|---|---|---|
| MA150713 | 14573 | IAEA C3 129,41 pmC | -2,074±19 | 1.29465 ±0.00304 |
| MA150713 | 24368 | B, Neutral phosphate 1 | 47,793 ±739 | 0.00261 ±0.00024 |
| MA150713 | 24369 | B, Neutral phosphate 2 | 50,992 ±972 | 0.00175 ±0.00021 |
| MA150713 | 24370 | B, No treatment 1 | 51,978 ±1,105 | 0.00155 ±0.00021 |
| MA150713 | 24371 | B, No treatment 2 | 53,261 ±1,303 | 0.00132 ±0.00021 |
| MA150713 | 11 samples/measurements not connected to this paper ($F^{14}C$ >0.59 for all) | | | |
| MA150713 | 24626 | Phthalic acid 070715 | 53,088 ±854 | 0.00135 ±0.00014 |
| MA150713 | 24626 | Phthalic acid 070715 | 55,091 ±785 | 0.00105 ±0.00010 |
| MA160114 | 26246 | Phthalic acid 101215 | 52,669 ±668 | 0.00142 ±0.00012 |
| MA160114 | 26350 | A, No treatment 1 | 362 ±19 | 0.95465 ±0.00227 |
| MA160114 | 26351 | A, No treatment 2 | 335 ±20 | 0.95862 ±0.00233 |
| MA160114 | 26352 | A, EDTA 1 | 329 ±19 | 0.95864 ±0.00228 |
| MA160114 | 26353 | A, EDTA 2 | 318 ±21 | 0.96045 ±0.00242 |
| MA160114 | 26354 | A, Neutral phosphate 1 | 316 ±21 | 0.96086 ±0.00245 |
| MA160114 | 26355 | A, Neutral phosphate 2 | 340 ±20 | 0.95753 ±0.00240 |
| MA160114 | 26356 | A, Acidic phosphate 1 | 307 ±21 | 0.96229 ±0.00247 |
| MA160114 | 26357 | A, Acidic phosphate 2 | 331 ±21 | 0.95891 ±0.00243 |
| MA160114 | 26358 | Background bone | 50,102 ±892 | 0.00195 ±0.00022 |
| MA160114 | 26601 | Phthalic acid 221215 | 51,373 ±612 | 0.00167 ±0.00013 |
| MA160114 | 26601 | Phthalic acid 221215 | 51,454 ±606 | 0.00165 ±0.00012 |
